# Parading towards healthier lives, the Mangyan of Mindoro

**DOI:** 10.3402/gha.v9.33863

**Published:** 2016-11-07

**Authors:** Alvona Zi Hui Loh

**Affiliations:** Yong Loo Lin School of Medicine, National University of Singapore, Singapore, Email: a0102161@u.nus.edu

**Figure:**
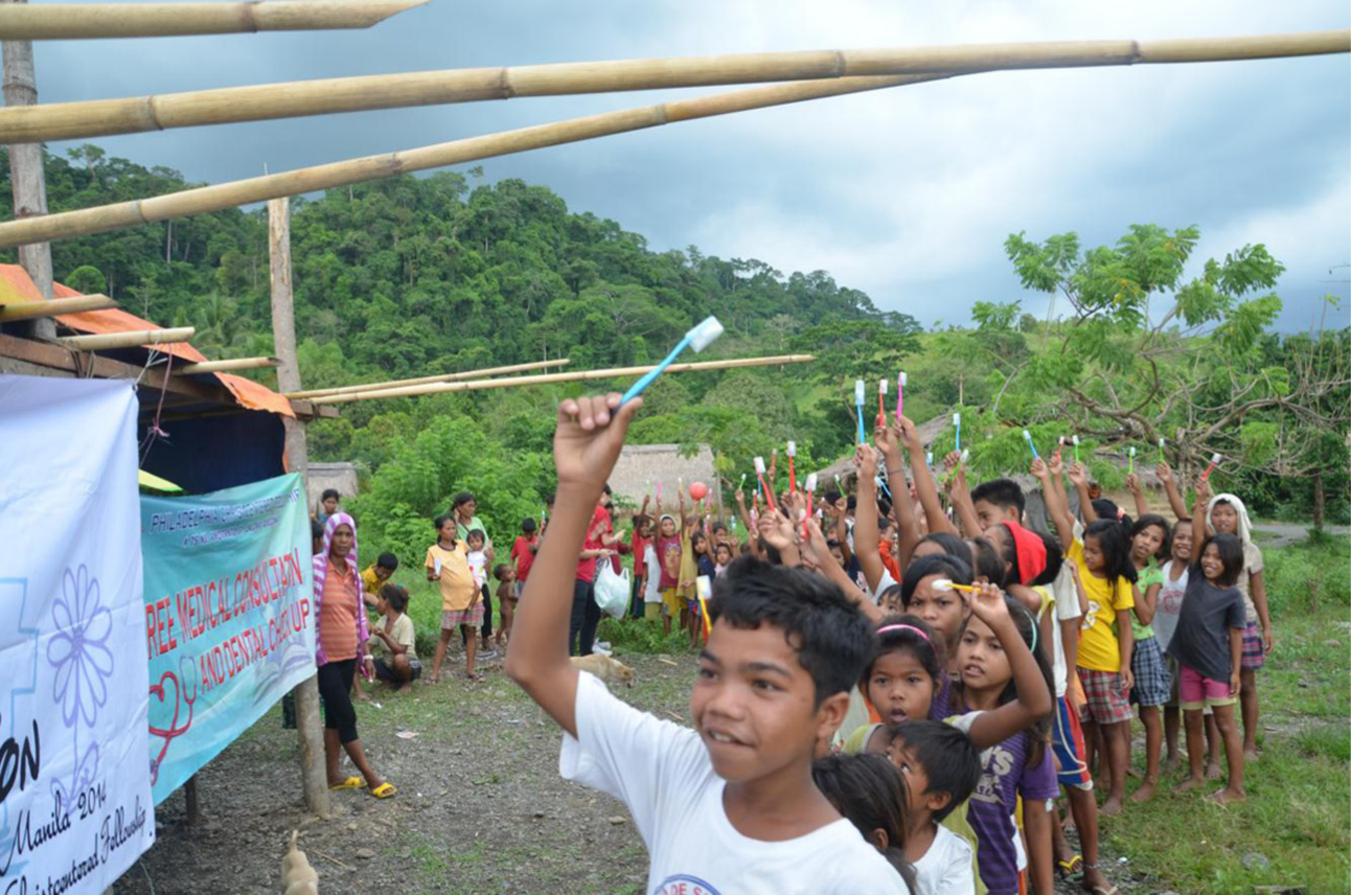
Photographer and Affiliation: Reon Yew Zhou Chin, Yong Loo Lin School of Medicine, National University of Singapore, Singapore

Global health aid provided by medical students in Singapore to vulnerable populations in Asia includes efforts in preventive medicine, for example, administration of vaccines and installation of water tanks, health education to improve knowledge on diseases afflicting communities, health screening, and affordable treatment for specific conditions.

The Manila Medical Mission is a student group, which provides humanitarian aid to tribes and villagers who live in Manila. They reach out to the Mangyan community, which consists of eight tribes in 17 villages on the island of Mindoro. Living in areas distant from town, this population lacks basic necessities such as clean water, shelters, and medicine. Many of them live in unsanitary environments and do not have basic health knowledge, resulting in problems such as poor sanitation and malnutrition.

In an effort to improve health conditions of the Mangyan community, provision of free medical aid, for example, wound dressing, health education, and distribution of toothbrushes to the tribes, was performed in June 2015. After obtaining their toothbrushes, the Mangyan community paraded around their village, raising their toothbrushes triumphantly as a sign of empowerment to lead healthier and better lives.

Empowering communities with knowledge to improve their health is rewarding, but is also a challenging process. We found that barriers to global health aid by medical students include difficulties in obtaining resources, such as funding and volunteers, as well as cultural and language barriers in foreign countries. However, solutions such as appropriate mentorship and sustainability plans that are project-specific can be considered to improve population health in the long run.

The photo is provided by Manila Medical Mission, a student group comprising primarily of medical students from Yong Loo Lin School of Medicine, National University of Singapore, who provide humanitarian aid to tribes and villagers who live in Manila.

Read Alvona Zi Hui Loh's other works in *Global Health Action*:

Loh AZ, Tan JS, Lee JJ, Koh GC. Voluntary community service in medical school: a qualitative study on obstacles faced by student leaders and potential solutions. Global Health Action. 2015; 8: 27562, doi: http://dx.doi.org/10.3402/gha.v8.27562

*Alvona Zi Hui Loh* Yong Loo Lin School of Medicine National University of Singapore Singapore Email: a0102161@u.nus.edu

